# Supplementary material for: Spectral cues are necessary to encode azimuthal auditory space in the mouse superior colliculus
Source: Nat Commun. 2020 Feb 27;11:1087. doi: 10.1038/s41467-020-14897-7 (PMC7046730; doi:10.1038/s41467-020-14897-7)
Supplement: Supplementary file 3 — Reporting Summary [file 41467_2020_14897_MOESM3_ESM.pdf]

nature research

Corresponding author(s): Shinya Ito
Alan M. Litke

Last updated by author(s): Jan 28, 2020

Reporting Summary

Nature Research wishes to improve the reproducibility of the work that we publish. This form provides structure for consistency and transparency in reporting. For further information on Nature Research policies, see Authors & Referees and the Editorial Policy Checklist .

Statistics

For all statistical analyses, confirm that the following items are present in the figure legend, table legend, main text, or Methods section.

n/a	Confirmed	
☐	☒	The exact sample size (n) for each experimental group/condition, given as a discrete number and unit of measurement
☐	☒	A statement on whether measurements were taken from distinct samples or whether the same sample was measured repeatedly
☐	☒	The statistical test(s) used AND whether they are one- or two-sided<br>*Only common tests should be described solely by name; describe more complex techniques in the Methods section.*
☒	☐	A description of all covariates tested
☐	☒	A description of any assumptions or corrections, such as tests of normality and adjustment for multiple comparisons
☐	☒	A full description of the statistical parameters including central tendency (e.g. means) or other basic estimates (e.g. regression coefficient) AND variation (e.g. standard deviation) or associated estimates of uncertainty (e.g. confidence intervals)
☐	☒	For null hypothesis testing, the test statistic (e.g. F, t, r) with confidence intervals, effect sizes, degrees of freedom and P value noted<br>*Give P values as exact values whenever suitable.*
☒	☐	For Bayesian analysis, information on the choice of priors and Markov chain Monte Carlo settings
☒	☐	For hierarchical and complex designs, identification of the appropriate level for tests and full reporting of outcomes
☐	☒	Estimates of effect sizes (e.g. Cohen's d, Pearson's r), indicating how they were calculated

Our web collection on statistics for biologists contains articles on many of the points above.

Software and code

Policy information about availability of computer code

Data collection	We used custom Matlab/LabView/Python/Arduino software to collect data.
Data analysis	We used custom Java/Matlab/Python software to analyze the data.

For manuscripts utilizing custom algorithms or software that are central to the research but not yet described in published literature, software must be made available to editors/reviewers. We strongly encourage code deposition in a community repository (e.g. GitHub). See the Nature Research guidelines for submitting code & software for further information.

Data

Policy information about availability of data

All manuscripts must include a data availability statement. This statement should provide the following information, where applicable:

- Accession codes, unique identifiers, or web links for publicly available datasets
- A list of figures that have associated raw data
- A description of any restrictions on data availability

The data that support the findings of this study are available from the corresponding author upon request.

Field-specific reporting

Please select the one below that is the best fit for your research. If you are not sure, read the appropriate sections before making your selection.

☒ Life sciences ☐ Behavioural & social sciences ☐ Ecological, evolutionary & environmental sciences

For a reference copy of the document with all sections, see nature.com/documents/nr-reporting-summary-flat.pdf

Life sciences study design

All studies must disclose on these points even when the disclosure is negative.

Sample size	We used blind analysis to determine the sample size. First, we hid half of the data and performed analysis on them. Once we reached to sufficient sample size to assess conclusions using the first half of the data (for each major finding in the data, we tried to achieve $p < 0.01$ in the exploratory dataset only), the other half was revealed to test the result. The rationale for the sufficiency of the sample size is granted by unblinding the data. If the blinded part of the data does not show significance, it could indicate an insufficient sample size. The exact number of mice that we used for each experiment depended on how quickly we could conclude the results in the exploratory dataset at $p < 0.01$.
Data exclusions	Data from a mouse were excluded if a recording from the mouse results in less than 10 neurons with an auditory receptive field (see text for details). This threshold was determined after ~10 initial experiments in which we identified the auditory map of space. Typical experiment yielded ~20 spatially localized neurons, with standard deviation of ~10 neurons. Once the number of neurons becomes less than 10, it is difficult to assess whether there is a map using a single mouse. Therefore, we set the 10 to be the threshold for data exclusion. This criterion was set in the middle of data collection for this study (at a point when we collected approximately 30% of the entire dataset for this study).
Replication	We used blind analysis to assess reproducibility of the data. The second half of the data was revealed after deciding features to look at. Therefore, we had done a complete replication of the entire experiment 'once' using the blinded dataset. The replication was successful except for the results for the mediolateral auditory map (please see Supplementary Fig. 5).
Randomization	Randomization is not applicable in our study because we only used wildtype CBA/CaJ mice.
Blinding	Blinding to group allocation is not applicable in our study because we only used wildtype CBA/CaJ mice.

Reporting for specific materials, systems and methods

We require information from authors about some types of materials, experimental systems and methods used in many studies. Here, indicate whether each material, system or method listed is relevant to your study. If you are not sure if a list item applies to your research, read the appropriate section before selecting a response.

Materials & experimental systems

n/a	Involved in the study	
☒	☐	Antibodies
☒	☐	Eukaryotic cell lines
☒	☐	Palaeontology
☐	☒	Animals and other organisms
☒	☐	Human research participants
☒	☐	Clinical data

Methods

n/a	Involved in the study	
☒	☐	ChIP-seq
☒	☐	Flow cytometry
☒	☐	MRI-based neuroimaging

Animals and other organisms

Policy information about studies involving animals; ARRIVE guidelines recommended for reporting animal research

Laboratory animals	We used 2–5 month-old CBA/CaJ (The Jackson Laboratory, 000654) mice of each sex.
Wild animals	This study did not involve wild animals.
Field-collected samples	This study did not involve field-collected samples.
Ethics oversight	All procedures were performed in accordance with the University of California, Santa Cruz (UCSC) Institutional Animal Care and Use Committee.

Note that full information on the approval of the study protocol must also be provided in the manuscript.
